# Synthesis and Antimicrobial Evaluation of 4*H*-Pyrans and Schiff Bases Fused 4*H*-Pyran Derivatives as Inhibitors of *Mycobacterium bovis* (BCG)

**Published:** 2018

**Authors:** Seyedeh Mahbobeh Mahdavi, Azizollah Habibi, Hadi Dolati, Seyed Mohammad Shahcheragh, Soroush Sardari, Parisa Azerang

**Affiliations:** a *Department of Organic Chemistry, Faculty of Chemistry,* *Kharazmi University, Tehran, Iran. *; b *Phytochemistry Research Center, Department of Pharmacognosy, School of Pharmacy, Shahid Beheshti University of Medical Sciences, Tehran, Iran.*; c *Drug Design and Bioinformatics Unit, Department of Medical Biotechnology, Biotechnology Research Center, Pasteur Institute of Iran, Tehran, Iran*.

**Keywords:** Antifungal, Anti-Mycobacterium, 4H-Pyran, N-Methylmorpholine, Schiff bases

## Abstract

The focus of our study is the synthesis and biological activity evaluation of a series of 4*H*-Pyran compounds and schiff bases fused 4*H*-Pyran derivatives which are known to possess a wide variety of biological activities. In this paper at first a simple and efficient one-pot synthesis of 4*H*-Pyran s from the three-component reaction between malononitrile, aldehydes, and active methylene compounds in the presence of *N*-methylmorpholine (NMM) as catalyst at room temperature is reported, the reaction between these synthesized products and trimethylorthoformateor triethylorthoformateto produce schiff base compounds were also considered. The key advantages of synthesis of 4*H*-Pyran derivatives are short reaction time, high yield, and simple work-up. Then, these compounds were evaluated for anti-Mycobacterium activity against *Mycobacterium bovis *(Bacillus Calmette–Guerin). The preliminary results indicated that most of the tested compounds showed relatively good activity against the test organism. Moreover, antifungal activities of these compounds were evaluated. Finally, their effect was more noticeable on *Mycobacterium bovis* (BCG).

## Introduction

Infectious diseases remain the largest cause of death in the world today, greater than cardiovascular disease or cancer (1). Based on an estimate by the World Health Organization (WHO) about two million people worldwide are infected with *Mycobacterium tuberculosis* (TB) each year and the number of new cases of tuberculosis has increased in recent years (2). Because of this, researchers showed interest in discovering new leads and active compounds effective against *Mycobacterium *species (3, 4).

Multicomponent reactions (MCRs) are special types of synthetically useful organic reactions in which three or more different starting materials react to give a final product in a one-pot protocol. Most of these reactions are atom-efficient processes by incorporating the essential parts of the starting materials into the final product. Major applications of MCRs described until today arise from the area of drug discovery (5, 6).

4*H*-Pyran s belong to an important class of heterocyclic compounds due to their wide biological and pharmaceutical properties, such as diuretic, spasmolytic, anticancer, anti-coagulant (7), antimicrobial (8), mutagenicity (9), antitumor (10), antiviral (11, 12), sex pheromone (13), anti-proliferative (14). Furthermore, these compounds can be used for the treatment of schizophrenia, alzheimer’s disease and mycolonous diseases (15). In addition, 4*H*-Pyran is a constituent of some natural products (16, 17) and a number of 2-amino-4*H*-Pyrans are useful as photoactive materials (18). Also, the antifungal and anti-TB activities of some 4*H*-Pyrans with different substitutions as a novel pharmacophore have also been investigated (19, 20).

On the other hand, schiff bases are compounds containing C=N group and interpret an important category of organic compounds, particularly in the medical and pharmaceutical fields due to a wide spectrum of biological activities like fungicidal, anticancer, antibacterial, herbicidal activities (21) antiviral (22), anti-inflammatory (23), anti-HIV (24) and antioxidant property (25). Additionally, schiff bases containing heterocycles possess excellent biological activities such as fungicidal, anticancer, antiviral, anti-HIV and bactericidal (26).

**Figure 1:**
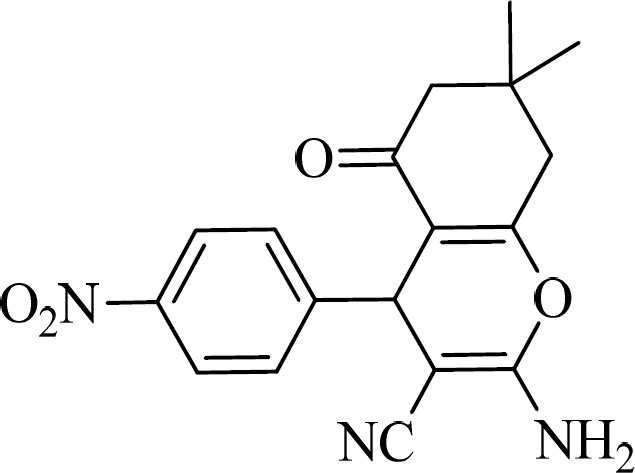
The structure of 2-amino-4*H*-Pyran compound with antibacterial activity

The antibacterial properties of some compounds derived from 2-amino-4*H*-Pyran (Figure 1) (9) prompted us to undertake the synthesis of various novels schiff bases derived from 4*H*-Pyran derivatives with the aim of improving activity.

The main purpose of this project is to develop synthetic methods in heterocyclic chemistry via Multicomponent Reactions (MCRs); specifically in schiff bases fused 4*H*-Pyran derivatives and especially to evaluate their anti-TB and antifungal activities. 

## Experimental

*General*

Melting points were measured using a capillary tube method with a Barnstead Electrothermal 9200 apparatus. FTIR spectra were recorded using KBr disks on Perkin-Elmer Spectrum RXI FT-IR Spectrophotometer. ^1^H and ^13^C NMR spectra were recorded on a Bruker AMX spectrometer at 300 MHz. The elemental analysis was performed with an Elemetar Analysensystem GmbH Perkin Elmer.

*General procedure for the preparation of 2-amino-4H-Pyran derivatives*

NMM (30 mol%) was added to a mixture of aldehydes (1 mmol), malononitrile (1 mmol) and 1, 3-diketone compounds (1 mmol) in ethanol (5 mL). The reaction was stirred at room temperature. After completion of the reaction, as indicated by TLC, the solid product was collected by filtration and purified by washing with water.

*General procedure for the preparation of Schiff base derivatives*

In a 10 mL round bottom flask, a mixture of 2*-*amino*-*4H*-*Pyran (1 mmol) and (trimethylorthoformate or triethylorthoformate) (2 mL) were placed over a magnetic stirrer and the contents were stirred and then 5 drops of acetic anhydride was added to this stirred mixture. The reaction mixture was heated under reflux for 7-9 h. The progress of the reaction was monitored by TLC. After completion of the reaction, the reaction mixture was evaporated under reduced pressure. The residue was dissolved in ethanol and water was added to the mixture and was stirred. The solid was collected by filtration, washed with water to obtain pure products. 

*In-vitro evaluation of anti-mycobacterial activity*

The broth microtiter dilution method against BCG (1173P2) has been used for *in-vitro* anti-mycobacterial activity evaluations of the compounds. Ethambutol and isoniazid were used as standard controls.

At first the test compounds were dissolved in DMSO to give a concentration of 1 or 2 mg/mL. All the wells of micro plates received 100 μL of freshly prepared Middle Broke 7H9 medium (Himedia, India), except for the first column. The medium in the test wells during incubation, 100 μL was added to the first column of 96-well plates. Then 100 μL of test compounds with desired concentrations (1000 or 2000 μg/mL) were added to the wells of the first row and serial double dilution was made from the first row to the last (each concentration was assayed in duplicate). Microbial suspension equal to 100 μL of BCG (1173P2), which had been prepared with standard concentration of 0.5 Mcfarland and diluted with 1:10 proportion by the distilled water, was added to all test wells. Incubation has was done for 96 h at 37 °C. Then, 12 μL Tween 80 10% and 20 μL Alamar blue 0.01% (Himedia, India) were added to each test well. The results were assessed after 24 and 48 h. The change to pink color against blue color is interpreted as a sign of bacterial growth. The MIC (minimum inhibitory concentration) is defined as the lowest drug concentration inhibiting color change from blue to pink. Ethambutol and isoniazid (Irandaru, Tehran) were used as positive and DMSO as negative control (27).

*Antifungal screening*

*Candida albicans* ATCC 10231 was used as test strain. The compounds were dissolved in dimethyl sulfoxide (DMSO) to reach concentration of 10 mg/mL. Antifungal activity tests performed by broth microdilution method. The absorbance was read at 530 nm in order to yield the desired transmittance of 75 to 77%. Working fungal culture was prepared from the stock fungal culture, a 1:1000 dilution with broth (*e.g.* 10 μL stock fungal culture: 10 μL broth) was prepared. Sabouraud maltose broth (DIFCO, Becton, Dickinson, USA) was used as the growth medium. Modified antimicrobial susceptibility testing is based on NCCLS M27-A method (28). Firstly, 100 μL of the broth was added to all wells of microplate, after that 40 μL of the test compounds and again 60 μL broth were added to well A. Secondly, a 100 μL solution serially diluted from well A by taking 100 μL into well B was obtained. This two-fold dilution was continued down the plate and 100 μL from the last well (H) was discarded. Finally, all the wells were filled with 100 μL of working fungal culture. Ketoconazole and amphotericin B (AmB) were used as a reference in the antifungal test. Wells containing serial dilution of DMSO and broth were prepared as control tests. The plate was sealed and incubated at 37 °C for 24 to 48 h. The minimum inhibitory concentration (MIC) values were obtained by reading the lowest concentration of compound in the well showing no growth.

*5-acetyl-2-amino-4-(3-methoxyphenyl)-6-methyl-4H-Pyran -3-carbonitrile (compound 4h)*

Yellow powder, mp: 157-160 °C. IR (KBr) (λ _max_, cm^-1^): 3334, 3185, 2193, 1676. ^1^H NMR (DMSO-*d*_6_, 300 MHz): δ 2.05 (s, 3H, CH_3_), 2.22 (s, 3H, CH_3_), 3.71 (s, 3H, CH_3_), 4.43 (s, 1H, CH), 6.69-6.82 (m, 3H, ArH), 6.87 (s, 2H, NH_2_), 7.22-7.27 (m, 1H, ArH). ^13^C NMR (DMSO-*d*_6_, 75.0 MHz): δ 18.4, 29.8, 55.0, 57.6, 111.7, 113.3, 114.8, 119.3, 119.8, 129.9, 146.2, 154.8, 158.3, 159.4, 198.4. Anal. calcd. for C_16_H_16_N_2_O_3_ (284.31): C, 67.59; H, 5.67; N, 9.85. Found: C, 67.44; H, 5.75; N, 9.77.

*5-acetyl-2-amino-4-(2-chlorophenyl) -6-methyl-4H-Pyran -3-carbonitrile (compound 4i)*

Cream powder, mp: 130-132 °C. IR (KBr) (λ _max_, cm^-1^): 3320, 3198, 2195, 1688, 1658. ^1^H NMR (DMSO-*d*_6_, 300 MHz): δ2.04 (s, 3H, CH_3_), 2.24 (s, 3H, CH_3_), 4.96 (s, 1H, CH), 6.93 (s, 2H, NH_2_), 7.19-7.43 (m, 4H, ArH). ^13^C NMR (DMSO-*d*_6_, 75.0 MHz): δ 18.6, 29.6, 44.7, 56.2, 114.2, 119.3, 128.1, 128.8, 129.6, 130.0, 131.7, 141.5, 155.7, 158.6, 197.9. Anal. calcd. for C_15_H_13_ClN_2_O_2_ (288.73): C, 62.40; H, 4.54; N, 9.70. Found: C, 62.24; H, 4.57; N, 9.65.

*MethylN-(3-cyano-7,7-dimethyl-4-(4-nitrophenyl)-5-oxo-5,6,7,8-tetrahydro-4H-chromen-2-yl) formimidate (compound 5a)*

Cream powder, mp: 168-171 °C. IR (KBr) (λ _max_, cm^-1^): 2963, 2212, 1661, 1612.^ 1^H NMR (DMSO-d_6_, 300 MHz): δ 0.97 (s, 3H), 1.04 (s, 3H), 2.20 (d, J=16.1 Hz, 1H), 2.27 (d, J=16.1 Hz, 1H), 2.59 (s, 2H), 3.85 (s, 3H), 4.65 (s, 1H), 7.56 (d, J=8.7 Hz, 2H), 8.19 (d, J=8.7, 2H), 8.57 (s, 1H). ^13^C NMR (DMSO-d_6_, 75.0 MHz): δ 27.1, 28.1, 31.9, 36.9, 49.9, 55.0, 81.4, 110.7, 116.9, 123.8, 129.3, 146.7, 150.1, 156.4, 162.9, 163.5, 195.8. Anal. calcd. for C_20_H_19_N_3_O_5_ (381.38): C, 62.99; H, 5.02; N, 11.02. Found: C, 63.03; H, 4.87; N, 10.92.

*Methyl N-(3-cyano-7,7-dimethyl-4-(3- nitrophenyl)-5-oxo-5,6,7,8-tetrahydro-4H-chromen-2-yl) formimidate (compound 5b)*

Cream powder, mp: 152-155 °C. IR (KBr) (λ _max_, cm^-1^): 2967, 2207, 1670, 1619. ^1^H NMR (DMSO-*d*_6_, 300 MHz): δ 0.98 (s, 3H), 1.05 (s, 3H), 2.14 (d, J=16.1 Hz, 1H), 2.27 (d, J=16.1 Hz, 1H), 2.60 (s, 2H), 3.30 (s, 3H), 4.7 (s, 1H), 7.65 (t, J=7.8 Hz, 1H), 7.76 (d, J=7.7 Hz, 1H), 8.08-8.13 (m, 2H), 8.62 (s, 1H). ^13^C NMR (DMSO-*d*_6_, 75.0 MHz): δ 26.9, 28.2, 32.0, 36.7, 49.9, 55.0, 81.6, 110.7, 116.9, 122.3, 122.4, 130.2, 134.7, 145.0, 147.9, 156.4, 162.9, 163.6, 195.8. Anal. calcd. for C_20_H_19_N_3_O_5_ (381.38): C, 62.99; H, 5.02; N, 11.02. Found: C, 63.10; H, 5.06; N, 10.99.

*Methyl N-(3-cyano-4-(4-methoxyphenyl)7,7-dimethyl-5-oxo-5,6,7,8-tetrahydro-4H-chromen-2-yl) formimidate (compound5c)*

Cream powder, mp: 162-163 °C. IR (KBr) (λ _max_, cm^-1^): 1956, 2209, 1667, 1612.^ 1^H NMR (DMSO-*d*_6_, 300 MHz): δ 0.96 (s, 3H), 1.03 (s, 3H), 2.12 (d, J=16.1 Hz, 1H), 2.25 (d, J=16.1 Hz, 1H), 2.56 (s, 2H), 3.71 (s, 3H), 3.83 (s, 3H), 3.84 (s, 1H), 6.86 (d, J=8.6 Hz, 2H), 7.13 (d, J=8.6, 2H), 8.57 (s, 1H). ^13^C NMR (DMSO-*d*_6_, 75.0 MHz): δ 26.9, 28.3, 31.9, 36.3, 50.0, 54.8, 55.0, 82.9, 111.8, 113.9, 117.2, 128.8, 134.8, 155.6, 158.3, 162.2, 162.6, 195.7. Anal. calcd. for C_21_H_22_N_2_O_4_ (366.41): C, 68.84; H, 6.05; N, 7.65. Found: C, 68.98; H, 6.07; N, 7.44.

*Methyl N-(3-cyano-4-(3-methoxyphenyl)7,7-dimethyl-5-oxo-5,6,7,8-tetrahydro-4H-chromen-2-yl) formimidate (compound 5d)*

Cream powder, mp: 148-150 °C. IR (KBr) (λ _max_, cm^-1^): 2964, 2212, 1662, 1610. ^1^H NMR (DMSO-*d*_6_, 300 MHz): δ 0.98 (s, 3H), 1.02 (s, 3H), 2.14 (d, J=16.1 Hz, 1H), 2.26 (d, J=16.1 Hz, 1H), 2.58 (s, 2H), 3.72 (s, 3H), 3.84 (s, 3H), 4.37 (s, 1H), 6.77 (t, J=8.4 Hz, 1H), 6.82 (d, J=2.1 Hz, 1H), 7.21-7.27 (m, 2H), 8.57 (s, 1H). ^13^C NMR (DMSO-*d*_6_, 75.0 MHz): δ 26.9, 28.2, 31.9, 37.1, 50.0, 54.9, 82.7, 111.5, 117.1, 127.2, 127.7, 128.5, 142.7, 155.8, 162.3, 162.9, 195.7. Anal. calcd. for C_21_H_22_N_2_O_4_ (366.41): C, 68.84; H, 6.05; N, 7.65. Found: C, 68.69; H, 6.12; N, 7.61.

*Ethyl N-(4-(4-chlorophenyl)-3-cyano7,7-dimethyl-5-oxo-5,6,7,8-tetrahydro-4H-chromen-2-yl) formimidate (compound 5e)*

Cream powder, mp: 167-170 °C. IR (KBr) (λ _max_, cm^-1^): 2209, 1663, 1605.^ 1^H NMR (DMSO-*d*_6_, 300 MHz): δ 1.05 (s, 3H), 1.13 (s, 3H), 1.38 (t, J=7.1 Hz, 3H), 2.39 (s, 2H), 2.49 (s, 2H), 4.40 (q, J=7.2 Hz, 3H), 4.51 (s, 1H), 7.20-7.30 (m, 4H), 8.25 (s, 1H). ^13^C NMR (DMSO-*d*_6_, 75.0MHz): δ 13.8, 27.6, 28.9, 32.3, 37.1, 40.7, 50.6, 64.5, 83.5, 112.6, 116.9, 128.9, 129.3, 133.3, 140.6, 155.9, 159.3, 162.3, 195.8. Anal. calcd. for C_21_H_21_ClN_2_O_3_ (384.86): C, 65.54; H, 5.50; N, 7.28. Found: C, 65.38; H, 5.49; N, 7.16.

*Methyl N-(4-(2-chlorophenyl)-3-cyano7,7-dimethyl-5-oxo-5,6,7,8-tetrahydro-4H-chromen-2-yl) formimidate (compound 5f)*

Cream powder, mp: 155-157 °C. IR (KBr) (λ _max_, cm^-1^): 2954, 2210, 1664, 1618.^ 1^H NMR (DMSO-*d*_6_*,* 300 MHz): δ 0.99 (s, 3H), 1.05 (s, 3H), 2.11 (d, J=16.1 Hz, 1H), 2.26 (d, J=16.1 Hz, 1H), 2.58 (s, 2H), 3.84 (s, 3H), 4.91 (s, 1H), 7.23-7.34 (m, 4H), 8.59 (s, 1H).^ 13^C NMR (DMSO-*d*_6_, 75.0 MHz): δ 27.0, 28.3, 31.9, 34.3, 40.9, 49.9, 54.9, 81.1, 110.8, 116.7, 127.7, 128.9, 129.6, 130.5, 132.4, 139.7, 156.2, 162.5, 163.6, 195.6. Anal. calcd. for C_20_H_19_ClN_2_O_3_ (370.83): C, 64.78; H, 5.16; N, 7.55. Found: C, 64.55; H, 5.08; N, 7.67.

*Methyl N-(3-cyano-7,7-dimethyl-5-oxo-4- phenyl-5,6,7,8-tetrahydro-4H-chromen-2-yl) formimidate (compound 5g)*

Cream powder, mp: 177-178 °C. IR (KBr) (λ _max_, cm^-1^): 2956, 2205, 1667, 1664. ^1^H NMR (DMSO-*d*_6_, 300 MHz): δ 0.97 (s, 3H), 1.04 (s, 3H), 2.13 (d, J=16.2 Hz, 1H), 2.26 (d, J=16.2 Hz, 1H), 2.58 (s, 2H), 3.84 (s, 3H), 4.40 (s, 1H), 7.21-7.34 (m, 5H), 8.58 (s, 1H). ^13^C NMR (DMSO-*d*_6_, 75.0 MHz): δ 26.9, 28.2, 31.9, 37.1, 50.0, 54.9, 82.7, 111.5, 117.1, 127.2, 127.7, 128.5, 142.7, 155.8, 162.3, 162.9, 195.7. Anal. calcd. for C_20_H_20_N_2_O_3_ (336.38): C, 71.41; H, 5.99; N, 8.33. Found: C, 71.24; H, 6.01; N, 8.30.

*Methyl N-(5-acetyl-3-cyano-4-(3-methoxyphenyl)-6-methyl-4H-Pyran -2-yl) formimidate (compound 5h)*

Cream powder, mp: 160-162 °C. IR (KBr) (λ _max_, cm^-1^): 2967, 2207, 1687. ^1^H NMR (DMSO-*d*_6_, 300 MHz): δ 2.09 (s, 3H), 2.29 (s, 3H), 3.73 (s, 3H), 3.82 (s, 3H), 4.70 (s, 1H), 6.78-6.88 (m, 3H), 7.29 (m, 1H), 8.55 (s, 1H). ^13^C NMR (DMSO-d_6_, 75.0 MHz): δ 18.3, 29.8, 54.8, 55.0, 81.8, 112.4, 113.7, 113.9, 117.2, 119.9, 130.1, 144.2, 155.5, 155.6, 159.5, 162.3, 198.1. Anal. calcd. for C_18_H_18_N_2_O_4_ (326.35): C, 66.25; H, 5.56; N, 8.58. Found: C, 66.15; H, 5.40; N, 8.70.

*Ethyl N-(5-acetyl-4-(2-chlorophenyl)-3-cyano-6-methyl-4H-Pyran -2-yl) formimidate (compound 5i)*

Cream powder, mp: 151-154 °C. IR (KBr) (λ _max_, cm^-1^): 2948, 2212, 1675, 1617. ^1^H NMR (DMSO-*d*_6_, 300 MHz): δ 1.35 (t, 3H), 2.07 (s, 3H), 2.33 (s, 3H), 4.37 (q, J=7 Hz, 2H), 5.24 (s, 1H), 7.19-7.28 (m, 3H), 7.38-7.42 (m, 1H), 8.22 (s, 1H). ^13^C NMR (DMSO-*d*_6_, 75.0 MHz): δ 13.8, 18.7, 29.3, 37.5, 64.3, 77.0, 77.4, 81.4, 113.3, 116.6, 128.0, 129.2, 130.2, 130.5, 132.9, 139.3, 155.9, 156.4, 159.5, 198.2. Anal. calcd. for C_18_H_17_ClN_2_O_3_ (344.79): C, 62.70; H, 4.97; N, 8.12. Found: C, 62.54; H, 5.01; N, 8.30.

## Results and Discussion

**Scheme 1:**
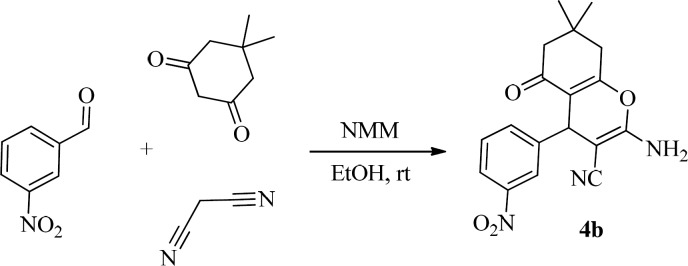
**Synthesis of** **schiff base compounds**

The new schiff bases were synthesized in two steps (Scheme 1). At first, 2*-*amino*-*4H*-*Pyran derivatives (4a-i)were synthesized according to a method with a three-component reaction comprising of 1,3-diketone (1), aldehydes (2) and malononitrile (3) in ethanol as solvent using *N*-methylmorpholine as an organocatalystat room temperature.

**Scheme 2:**
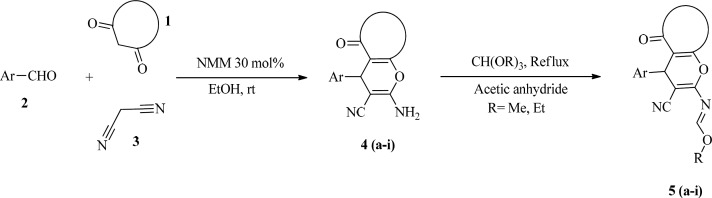
**Synthesis of 2-amino-4** ***H*** **-Pyran** **compounds**

The reaction of 3*-*Nitrobenzaldehyde, malononitrile and dimedone were employed as a template to optimize the reaction conditions (Scheme 2).

Therefore, a mixture of m*-*Nitrobenzaldehyde (1 mmol), malononitrile (1 mmol) and dimedone (1 mmol) in EtOH was stirred for an appropriate time as indicated by TLC using different amounts of NMM. At the end of reaction, the knoevenagel condensation between aldehydes and malononitrile followed with dimedone resulted in only one product called 4b. To show that NMM is an efficient catalyst, this three components› reaction was accomplished in the absence of catalyst at room temperature for 5 h. This reaction just produced the product of knoevenagel condensation between aldehydes and malononitrile.

The efficiency of the reaction is mainly affected by the amount of the catalyst (Table 1). The optimal amount of the catalyst was 30 mol% (entry 4); the higher amount of the catalyst did not noticeably increase yield (entry 5).

**Table 1:** Screening of catalyst in the formation of 4b

**Entry**	**(Catalyst mol%)**	**Time (min) 4b**	**Yield (%) 4b**
1	(NMM 5 mol%)	10	83
2	(NMM 10 mol%)	5	86
3	(NMM 20 mol%)	3	87
4	(NMM 30 mol%)	2	90
5	(NMM 40 mol%)	3	88

To generalize this methodology with the optimal conditions, a variety of aldehydes and a series of 1,3-diketone compounds were used to prepare a wide range of 2-amino-4*H*-pyran compounds (Table 2), entries (4a-i) in excellent yields.

**Table 2:** **Synthesis of** **2** ***-*** **amino 4H-Pyran** **derivatives catalyzed by NMM**

**Entry**	**Ar**	**1, 3-diketone**	**Product**	**Time** **(min)**	**Yield%**	**Melting point (°C)**	
						**Found**	**Ref**
1	p-O_2_NC_6_H_4_	Dimedone	4a	10	95	184-187	185-186 (29)
2	m-O_2_NC_6_H_4_	Dimedone	4b	2	90	216-218	215-216 (29)
3	p-CH_3_OC_6_H_4_	Dimedone	4c	15	88	202-204	200-201 (29)
4	m-CH_3_OC_6_H_4_	Dimedone	4d	5	81	189-191	190-191 (29)
5	p-ClC_6_H_4_	Dimedone	4e	11	88	214-216	215-216 (29)
6	m-ClC_6_H_4_	Dimedone	4f	8	93	215-217	216-217 (29)
7	C_6_H_5_	Dimedone	4j	4	92	224-226	225-226 (29)
8	m-CH_3_OC_6_H_4_	Acetylacetone	4h	7	88	157-160	
9	o-ClC_6_H_4_	Acetylacetone	4i	9	88	140-142	

After synthesis of 2*-*amino*-*4H*-*Pyran, their schiff base derivatives (5a-i) were prepared by the condensation reaction between the amines (2*-*amino*-*4H*-*Pyran derivatives) and the aldehydes (trimethyl or triethyl) orthoformate under reflux conditions and catalytic amount of acetic anhydride. All reactions produced corresponding Schiff-bases (5a–i) in good yield; the results were summarized in Table 3.

**Table 3:** **Synthesis of** **Schiff-bases (** **5a-i** **)** **derivatives**

**Entry**	**Amine**	**Aldehydes**	**Product**	**Time (h)**	**Yield%**
1	4a	Trimethylorthoformate	5a	8.5	70
2	4b	Trimethylorthoformate	5b	7	73
3	4c	Trimethylorthoformate	5c	9	66
4	4d	Trimethylorthoformate	5d	7.5	61
5	4e	Triethylorthoformate	5e	8.5	62
6	4f	Trimethylorthoformate	5f	7.5	71
7	4j	Trimethylorthoformate	5j	8	69
8	4h	Trimethylorthoformate	5h	8	65
9	4i	Triethylorthoformate	5i	8.5	64

All the synthesized and purchased compounds were evaluated for anti-mycobacterial and antifungal activity; the results have been shown in Table 4.

**Table 4:** **Anti-Mycobacterium and antifungal activity expressed as MIC (μM)**

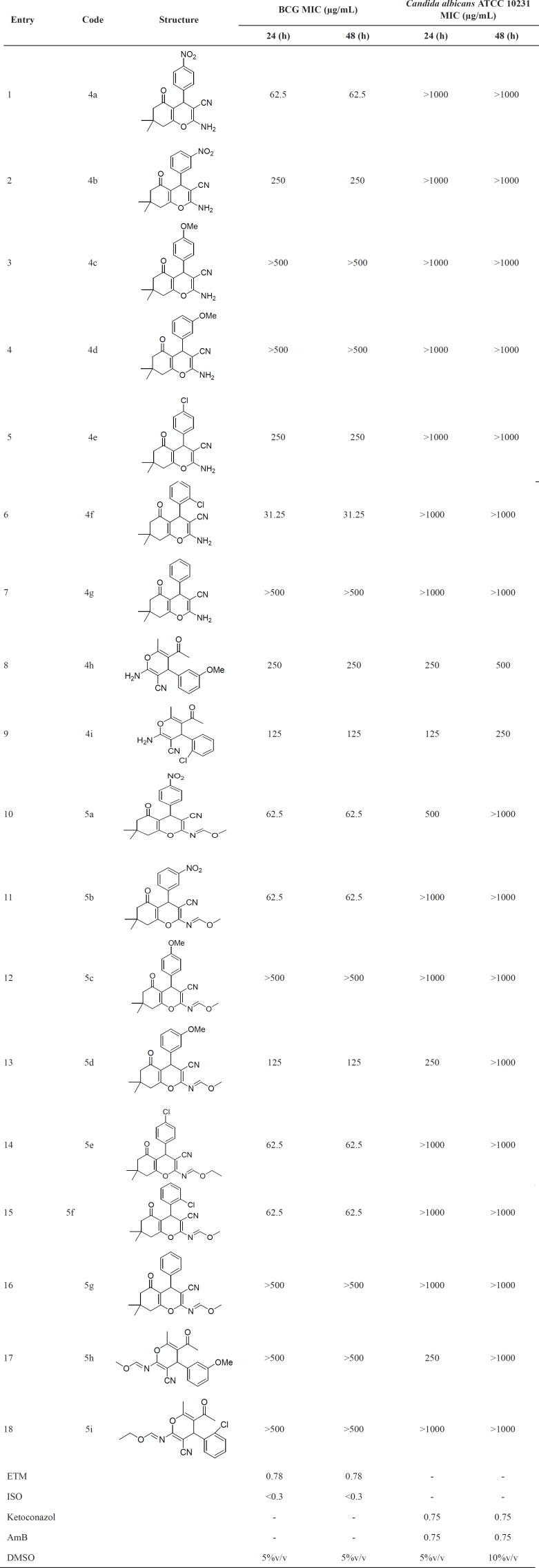

**MIC: Minimum Inhibitory Concentration; ETM: Ethambutol; ISO: Isoniazid; AMB:** **amphotericin B.**

As indicated in Table 4, the majority of the MIC results for the tested compounds showed a potentially good effect against *Mycobacterium* *bovis* (*M. bovis)*. The compound 4f showed the highest antimycobacterial activity (31.25 μg/mL) and compounds 4a, 5a, 5b, 5e, 5f demonstrated the high activity against *Mycobacterium *(62.5 μg/mL) (Table 4). It seems the activity of compounds depends on electron donor and also the position of substituents in aromatic ring. 

It is evident that the rings substituted with electron withdrawing groups such as NO_2_ and Cl showed better activity than donor substituent such as OMe in 4c or unsubstituted phenyl ring compound such as 4j and 5j compounds.

Presence of Cl substitution in 2-position in benzene ring (compound 4f) resulted in better activity against *Mycobacterium* than compound 4e which include Cl substitution in 4-position.

In schiff bases fused 4*H*-Pyran compounds, most of 4*H*-Pyran s which synthesized from dimedone such as 4a-4j, showed better anti-mycobacterial activity compared to those which were synthesized from acetylacetone such as 4h and 4i. Moreover, to assess antimicrobial activity of these compounds, antifungal activity was evaluated. Candida albicans (C.albicans) is introduced as a good candidate for antifungal activity tests. Mainly, the treated compounds showed poor activity against C.albicans, compounds 4h and 4i, which were synthesized from acetylacetone, scored the highest antifungal activity.

## Conclusion

In conclusion, a series of biologically and pharmacologically active 4*H*-Pyran derivatives (4a-i) have been synthesized under simple conditions and in the attendance of NMM as a catalyst, subsequently new high-yield Schiff-bases (5a-i) containing 4*H*-Pyran core synthesized. The anti-mycobacterial and antifungal test results demonstrated that the majority of the synthesized compounds were active against *M. bovis* and they have poor activity against *C.albicans*.
